# Stability Study of Sunscreens with Free and Encapsulated UV Filters Contained in Plastic Packaging

**DOI:** 10.3390/pharmaceutics9020019

**Published:** 2017-05-31

**Authors:** Benedetta Briasco, Priscilla Capra, Barbara Mannucci, Paola Perugini

**Affiliations:** 1 Department of Drug Sciences, University of Pavia, Pavia, PV 27100, Italy; benedetta.briasco@gmail.com (B.B.); priscilla.capra@unipv.it (P.C.); 2 C.G.S. (Centro Grandi Strumenti), University of Pavia, Pavia, PV 27100, Italy; barbara.mannucci@unipv.it; 3 Etichub s.r.l, Academic Spin-Off, Department of Drug Sciences, University of Pavia, Pavia, PV 27100, Italy

**Keywords:** sunscreens, stability, packaging, NIR, multiple light scattering technique

## Abstract

Sunscreens play a fundamental role in skin cancer prevention and in protection against photo-aging. UV filters are often photo-unstable, especially in relation to their vehicles and, being lipophilic substances, they are able to interact with plastic packaging. Finally, UV filter stability can be significantly affected by the routine use of the product at high temperatures. This work aims to study the stability of sunscreen formulations in polyethylene packaging. Butyl methoxydibenzoylmethane and octocrylene, both in a free form and as encapsulated filters were chosen as UV filters. Stability evaluations were performed both in the packaging and on the formulations. Moreover, a further two non-destructive techniques, near-infrared (NIR) spectroscopy and a multiple light scattering technique, were also used to evaluate the stability of the formulation. Results demonstrated clearly that all of the pack underwent significant changes in its elastic/plastic behavior and in external color after solar irradiation. From the evaluation of the extractable profile of untreated and treated packaging material an absorption of 2-phenoxyethanol and octocrylene were shown. In conclusion, the results highlighted clearly that a reduction of the UV filter in the formulation packed in high-density polyethylene/low-density polyethylene (HDPE/LDPE) material can occur over time, reducing the protective effect of the product when applied to the skin.

## 1. Introduction

The number of skin cancers diagnosed annually is growing year after year. It is well recognized that this kind of cancer is caused mainly by the negative effects of solar exposure, especially exposure to ultraviolet (UV) radiation [1]. UVB (280–320 nm) typically induces erythema and direct DNA damage via pyrimidine dimer formation, whereas UVA (320–400 nm) is associated with tanning, photo-aging and the generation of excess reactive oxygen species, which indirectly damage DNA [2,3,4]. For this reason, in order to reduce skin photo-damage and the carcinogenic effects of solar irradiation, the use of sunscreen products containing UV filters as an integral part of a photoprotection strategy has gained popularity [5].

An ideal sunscreen provides uniform protection against the UVB and UVA wavelength range, maintaining sensorial features that enhance the user’s experience. 

In order to absorb ultraviolet radiation (UVR), an organic molecule must contain a suitable chromophore presenting conjugated π-electron systems [6]. The absorption of a UV photon results in the molecule being energized to an excited electronic state. If the absorbed energy is not dissipated as heat, the chemical bonds of the molecule may break, resulting in a degradation of the UV filter. This phenomenon can transform the UV filter to another chemical entity with less protective efficacy and, sometimes, with unknown toxicity leading to a safety reduction of the final product [7]. 

Most of the UV absorbers used in sunscreens are photostable under foreseeable conditions of use. Two exceptions are butyl methoxydibenzoylmethane (avobenzone) and octinoxate [8]. In particular avobenzone undergoes rapid photodegradation, when used alone, and for this reason it is often stabilized by addition of the UV filter octocrylene [6].

In the literature reports on the photostability of UV filters indicate that analyses are made in diluted solutions, however, this is not relevant to a complex matrix, like sunscreen final products, composed of water in oil or oil in water emulsions, where the photochemistry of these filters is very different from those in a diluted solution. The behavior of sunscreen final products are also not predictable from the photostability of its individual filters, so it is important to evaluate the final formulation [9,10]. In fact, sunscreen vehicles can often impact on the sun protection factor (SPF) [11]. Thus, sunscreen efficacy and safety depend on the ultraviolet filter types (organic or inorganic), their photostability, the excipients used, and the choice of packaging.

Furthermore, the lipophilic nature of filters must be taken into account, with respect to their capability to be very good solvents against the packaging material. Thus, the choice of packaging is fundamental for a good quality product. 

Among the different kinds of materials used as the primary packaging, polyolefins and, especially, polyethylene (PE), are the most widely used plastics in sunscreen products. Polyethylene is classified into several categories based mostly on density and branching, with all polyethylenes being semicrystalline.

At present, many hundreds of grades of PE, most of which differ in their properties in one way or another, are available. PE also possesses good chemical stability. PE can be easily heat sealed, is tough, and has high elasticity. It has good cold resistance properties and is a good water vapor barrier. However, low-density polyethylene (LDPE) has low barrier properties to gases, aromas, and fats. With increasing density, all of the barrier properties increase, as well as the stiffness, hardness, and strength, as a result of the higher crystallinity. At the same time, there is a decrease in the impact resistance, toughness, resistance to stress cracking, cold resistance, and transparency.

LDPE has a gas permeability in the range normally expected with rubbery materials. High-density polyethylene (HDPE) has a permeability of about one-fifth that of LDPE. Polyethylene is cheap, and particularly easy to mold and fabricate. It accepts a wide range of colors, can be transparent, translucent, or opaque, has a pleasant, slightly waxy feel, can be textured or metal-coated, but is difficult to print on [12].

Furthermore, polyolefins are lipophilic materials, so they are able to retain large amounts of compounds with the same nature, like suncreens [13]. Moreover it is also necessary to take into account that these products are often stored in a very warm environment, so the entire system (formulation + package) must remain stable in these conditions [14]. Thus, the evaluation of the sunscreen formulation stability in the final packaging is important to assure the efficacy and the safety of the final product.

In view of these considerations, this work aims to study the stability of a sunscreen product, packed in a LDPE/HDPE mixture under conditions simulating the possible stress conditions encountered during their shelf life. 

Butyl methoxydibenzoylmethane (avobenzone) and octocrylene were chosen as UV filters. Avobenzone (4-tert-butyl-4′-methoxydibenzoylmethane) is one of the most common UVA filters in sunscreens. The photochemical behavior of this filter has been extensively studied and its photostability has been found to be highly dependent on the polarity and proticity of the solvent. In solution, avobenzone enol forms photoisomerized keto forms, revealing a great photoinstability [15,16,17,18]. 

Octocrylene is an ester formed by the condensation of diphenylcyanoacrylic acid with 2-ethylhexanol, and is considered to belong to the family of cinnamates. The action spectrum of octocrylene (290–360 nm, peak absorption at 303 nm) covers mostly UVB wavelengths, but also short UVA wavelengths (UVAII) [19]. Usually octocrylene is considered a stable filter, and it is also able to stabilize photo-unstable filters like avobenzone, but it is expensive and difficult to incorporate into sunscreens [15,20,21].

In this work these filters were used both in unencapsulated and encapsulated forms in sol-gel silica glass from commercially-available aqueous dispersions [22].

The encapsulation of organic UV filters should permit the increase of their UV stability and to be able to prevent both interactions between the substances and the skin and those between filters and the external environment (e.g., with the package).

Oil/water (O/W) emulsions were then prepared with both forms of UV filters maintaining the same ratio between the two filters and choosing excipients consistent with the commercial product.

In order to evaluate system stability, analyses were carried out both on packaged and unpackaged formulations. Containers underwent tensile testing, colorimetric assessment, and extractable testing. Sunscreen formulations were characterized in terms of pH, organoleptic properties, rheological behavior, and filter content. Moreover, a further two non-destructive techniques, including near-infrared spectroscopy (NIR) and the multiple light scattering technique, were used in order to determine the stability of the formulations.

## 2. Materials

Sucrathix VX by Alfa Chemicals (Berkshire, UK), glycerin and Ethylenediaminetetraacetic acid disodium salt EDTA by CARLO ERBA reagents (Cornaredo, Italy), Tegosoft TN by Evonik Industries (Essen, Germany), Montanov L and Montanov 82 by Seppic (Puteaux, France), Eusolex UV Pearls OB SX (octocrylene and butyl methoxydibenzoylmethane encapsulated in silica shells in a white viscous dispersion containing PVP and sorbitol), Eusolex OCR (Octocrylene) and Eusolex 902 (butyl methoxydibenzoylmethane) by Merck KGaA (Darmstadt, Germany), and Verstatil PC by Dr. Straetmans GmbH (Hamburg, Germany), were obtained and used unchanged.

The composition of Eusolex UV pearls were the following: water, octocrylene of about 30%, sorbitol, butylmethoxydibenzoylmethane of about 9%; silica and PVP (octocrylene, butylmethoxydibenzoylmethane, 3.5:1 *w*/*w*).

Ethanol (96% *v*/*v*) and other chemical reagents were obtained from CARLO ERBA reagents (Cornaredo, Italy).

Bottles of 125 mL made of a HDPE/LDPE mixture were used as the packaging material, they were a gift from an Italian supplier.

## 3. Methods

### 3.1. Formulation Preparation

For this study, three formulations were developed: the first (F1) represents the placebo (without UV filters); the second is the formulation containing the UV filters octocrylene and butyl methoxydibenzoylmethane (F2) in the free form, and the third contains encapsulated octocrylene and butyl methoxydibenzoylmethane (F3).

The compositions of these formulations are represented in Table 1. 

Excipients were chosen for those commonly used in topical formulations containing UV filters.

Antioxidants were not included into formulations to be able to detect any signs of UV filter chemical instability. 

Each formulation was prepared in triplicate by emulsification, slowly adding phase B into phase A, using a Silverson SL2T High Shear Laboratory Stirrer Mixer (Silverson Machines Ltd., Chesam, UK) at 7000 rpm for 10 min, at 75 °C. The O/W emulsion was then cooled and phase C was added, when the temperature was below 40 °C. Then formulations were packaged into LDPE/HDPE bottles, with some samples in glass bottles, as controls.

### 3.2. Stress Testing Procedures

All formulations maintained in LDPE/HDPE mixture bottles were subjected to two degradation tests, in order to simulate in-use stress conditions. In particular:

➢ A photostability test, by simulating solar irradiation using a SUNTEST XLS +II (Atlas^®^, Chicago, IL, USA) for 96 h. The SUNTEST instrument was set up according to standard European procedures [23], with the following parameters: two Solar ID 65 filters, a cycle of 96 h corresponding to 192 h solar light; irradiation control between 300–800 nm; 750 W/m^2^ irradiation potency; room temperature of 35 °C; and the black standard temperature (BST) set at 45 °C.
  In order to assure that both parts of each single dose container underwent simulated UV irradiation for the same period and under the same conditions, after 48 h containers were turned to expose the other side.
➢ Thermal cycles (20 °C for 36 h–45 °C for 12 h, three times) were performed using an BRE60 BioExpert 56 L incubator (Froilabo, Meyzieu, France).

At the end of both experiments the samples were taken, emptied, and the bottles were washed using a standard procedure. Briefly, all bottles were washed three times with 1% bicarbonate solution and then rinsed three times with distilled water to remove any residue. 

All formulations were also kept at room temperature (22 ± 2 °C) for three months, both in glass and in LDPE/HDPE bottles, in order to determine any variation with respect to formulations subjected to the stress conditions above, compared to standard room temperature.

### 3.3. Packaging Evaluation

Packaging stability was evaluated by mechanical, colorimetric, and extractable profile analyses, as reported below.

#### 3.3.1. Mechanical Test

The investigation of the mechanical properties of the containers was performed on each bottle, as reported in a previous work by Perugini et al. [24], using a tensile machine, AGS 500ND (Shimadzu corporation, Kyoto, Japan) equipped with a 500 N load cell and a strain rate of 10 mm/min.

For this purpose 20 samples for each type of container (empty and not treated filled, respectively, with the two formulations and treated with simulated solar irradiation and thermal shock as described above) were obtained. Briefly, “bone-shape” samples were obtained from the central part of each container, horizontally, according to the principles of the European Standard EN ISO 527 [25], suitably modified for a bottle, following previous work by this research group [24]. Specifically, this optimized dog bone shape obtained by punch-cutting provides a localized stress region (3 mm width) called the break point. Each sample was characterized for thickness and width of this region, using a BW 1008 digital microscope. The section of each sample was calculated for thickness and width using suitable software (Micromeasure vers.1.2, Colorado State University, Fort Collins, CO, USA). 

Samples were kept under constant temperature (23 ± 2 °C) and Relative Humidity (R.H.) (52 ± 5%.) for a week until tension tests started and during the entirety of the test time, to obtain a stress versus strain curve. From each set of results the tendency of materials to oppose deformation was estimated, and the curve profile in the elasticity regime, the elongation percentage in the elasticity regime, and the absolute elongation elasticity were evaluated.

A critical analysis and comparison of diagrams made of different specimens allows a first assessment of any significant changes in the stress-strain diagram due to interactions between the material they are made of and the conditions or substances which they are in contact with [24].

The data obtained from the mechanical test were processed by statistical analysis (Mann-Whitney test), at a confidence range of 95%, so the changes were considered statistically significant for *p* < 0.05.

#### 3.3.2. Colorimetric Analysis

For the colorimetric evaluation of the packaging, the measurement of the yellowness index (YI), following ASTM E313, was performed [26]. The yellowness index describes the color change of a test sample from clear or white to yellow.

For this purpose the instrumental assessment of packaging color was performed with a CL400 Colorimeter (Cutometer MPA580, CK Electronic GmbH, Cologne, Germany). Technical data of the probe are: length: 126 mm; illumination: Ø 24 mm; measuring area: Ø 8 mm; weight: 85 g; illuminated area: approx. 17 mm Ø; units: xyz, and RGB, L 3 a 3 b; light: eight white LEDs arranged circularly; range of emitted wavelengths: 440–670 nm; and accuracy: 65%.

The color acquisition was done by specifying the three tri-stimulus values X, Y, and Z, of a color in accordance with the CIE system, where X is the tri-stimulus value of red, Y is the green value, and Z is the blue value. With a mathematical equation it was possible to use these parameters in order to calculate the YI value [27].

#### 3.3.3. Testing of Extractable Substances

This step aims to evaluate the possible migration of substances from packaging, or the absorption of some ingredients from the formulation on contact between plastic containers and the prepared formulations after stress testing.

Packaging materials were exposed to harsh solvents and conditions to generate every potential extractable substance. The extraction technique selected for this purposed was the head space solid phase microextraction (HS-SPME), since HS-SPME extracts generally contained the same extractable substance as did the Soxhlet extracts, providing a complete insight into all of the predominant organic extractables [28].

The HS-SPME conditions set were: fiber: 100 µm polydimethylsiloxane (PDMS); Supelco; 500 mg sample, headspace mode; incubation temperature: 90 °C, extraction time: 60 min.

The resulting extracts were chemically characterized by gas chromatography-mass spectrometry (GC/MS). In Table 2 parameters of GC/MS system are reported. Analyses were carried out on a Thermo Scientific (Thermo Fisher Scientific, Waltham, MA, USA) GC/MS system (TraceDSQII mass spectrometer, TraceGCUltra gas chromatograph, and a CTC Analytics COMBIPAL autosampler) using Xcalibur MS software version 2.2. The mass spectra of the detected extractable compounds were compared with the GC/MS NIST Mass Spectral Library (NIST 08) and Wiley Registry of Mass Spectral Data 8th Edition databases. Although the databases were used, some classes of compounds, such as alkanes, yielded very similar fingerprint patterns or fragments and, thus, it was not always possible to make an indisputable identification of every peak (compound) detected.

### 3.4. Formulation Characterization

All formulations were characterized in terms of pH, and organoleptic and rheological properties, both after 24 h from preparation and after stress testing.

The pH measurement was performed by a Jenway 3510 pH meter (Jenway, Staffordshire, UK), pH 4 and 7 standard buffers were employed to calibrate the instrument before use.

Rheological properties were evaluated using a Brookfield viscometer, model RVT (Brookfield AMETEK, Middleboro, MA, USA). The sample (125 g) was placed in a glass pot and left to equilibrate for 15 min, before measuring with the RV 5 spindle at different speeds (0.5, 1, 2.5, 5, 10, 20, 50, and 100 rpm). The measurements were carried out under controlled conditions of temperature and humidity (23 °C; 52% RH), with viscosity values expressed in mPa·s. 

### 3.5. Assessment of UV Filter Content

The amount of free and encapsulated UV filters in the formulations was monitored using a UV-VIS spectrophotometer, model AGI-UV 8453 (Agilent, Santa Clara, CA, USA). Briefly, two calibration curves of octocrylene and butyl methoxydibenzoylmethane (avobenzone) were constructed by reading the absorbance at 303 (λ_max_ of octocrylene) and 361 nm (λ_max_ of avobenzone) corresponding to the maximum wavelengths of each filter. Four levels of diluted stock solutions in the concentration range of 5–30 and 2–15 μg/mL for octocrylene and avobenzone, respectively, starting from stock solutions of 2 mg/mL in ethanol were prepared. All samples were prepared in triplicate. In the preliminary phase a suitable HPLC method was also carried out in order to validate the UV method [29] using an HPLC, Agilent model 1100 (Agilent, Santa Clara, CA, USA), equipped with a Shiseido C18, 5 μm (250 × 4.6 mm) column. The compounds were eluted using a mobile phase of MeOH:H2O (90:10% *v*/*v*) at a flow rate of 1.0 mL/min and they were monitored using a UV detector at 320 nm. 

For the analysis of filter content in the formulations, stock solutions (500 mg of formulation in 100 mL ethanol) were prepared and left under stirring overnight to allow for the complete extraction of filters from the formulation. Then, they were filtered using PTFE syringe filters (0.45 µm porosity) with different dilutions were prepared in triplicate, and analyzed using UV spectrophotometry.

### 3.6. Near-Infrared (NIR) Analysis

The formulations were also analyzed using NIR spectroscopy for a further non-destructive characterization in order to reveal possible changes before and after treatments in formulations themselves. For the NIR spectroscopy, formulations were scanned in a range of the near-infrared region (950–1650 nm). In this region, each constituent of a complex organic mixture has unique absorption properties, due to the stretching and bending vibrations in molecular bonds [30]. 

For this analysis the MicroNIR™ Pro Spectrometer, (JDSU, Milpitas, CA, USA) was used, with parameters reported in Table 3. This technique presents some advantages with respect to other analytical techniques: for example, its ability to record the spectra of solid and liquid samples without prior manipulation. Furthermore, it is simple, rapid, and cost-effective [31]. 

Formulation samples were placed on a non-reflective support, with a fixed and constant distance (3 mm) from the acquisition window. Three replicates for each sample were always performed.

Data were evaluated using the Unscrambler^®^ X software, version 10.4 (Camo software AS, Oslo, Norway). Principal components analyses (PCA) on pretreated spectra were performed on the obtained spectra in order to determine the capability of the NIR measurement system to discriminate different samples and possible changes in formulations, due to treatments and/or aging. The spectra were pretreated by using standard normal variate (SNV) followed by the first derivative with Savitzy-Golay smoothing.

### 3.7. Multiple Light Scattering Analysis

The multiple light scattering technique was used for investigating the stability of the formulations after three months of storage. This technique is an optical method to characterize concentrated liquid dispersions without dilution [32,33]. Specifically, for this purpose, a Turbiscan MA 100 (Formulaction SA, L’Union, France), was used. The main advantage of the Turbiscan is the ability to detect particle size changes and/or local concentration changes, which result in variations of the backscattering (BS) and transmission (T) signals while the sample is destabilizing, in a more accurately and faster way than human eye can do.

The functional principle is to send photons (NIR light source, 880 nm) into the sample. The photons, after being scattered many times by the particles (or droplets) in the dispersion emerge from the sample and are detected by the two detectors of the Turbiscan reading head, detecting the transmission for non-opaque samples (0° from light source) and backscattering for opaque samples (135° from the light source). Backscattering is directly related to the photon transport mean free path, so backscattering intensity depends on particle size and concentration.

After different storage times the variation of the ΔT and ΔBS are measured as a function of the test tube height and compared to the initial transmission and backscattering profiles. A plot is produced of these results with ΔT or ΔBS on the y-axis and the sample height (h, mm) on the x axis. A sample height of 0 mm corresponds to the bottom of the measurement cell. A backscattering increase indicates droplet size changes, due to aggregation and coalescence, as well as sedimentation.

Finally, formulations with UV filters were compared using the Turbiscan stability index (TSI), which represents an absolute value related to the general behavior of the formulation; TSI summarizes all of the variations detected in the samples in terms of particle size and/or concentration. The higher the TSI, the higher the instability rate of the sample. In this work, TSI values at different heights of the sample and at different times were considered. Formulations were mixed and then transferred in test tubes (10 cm height) and stored at 20 ± 2 °C for three days. Samples were scanned (number of total scans: 216) for at least three days. 

## 4. Results and Discussion

This work aims to evaluate the stability of formulations containing UV filters and packed in a commonly-used plastic packaging (LDPE/HDPE mixture). Bottles made of an LDPE/HDPE mixture were chosen in this study for two reasons: first, it is a very common matrix for sunscreen products and, second, in a previous work the authors had already studied the influence of PE with different densities and branches of lipophilic content [34].

In the present work the influence of the physical appearance of UV filters, as in a free or in encapsulated form, in relation to the formulation stability, were investigated. Furthermore two different stress treatments (solar irradiation simulating test and thermal shock cycles), were carried out and their influence on packaging integrity and formulation stability were also studied.

### 4.1. Packaging Evaluation

#### 4.1.1. Mechanical Analysis

For the evaluation of possible changes in packaging due to treatments and/or contact with filling formulations, a tensile test was performed on the packaging in order to investigate the mechanical behavior of plastic containers, after exposure to stress conditions.

**Figure 1:**
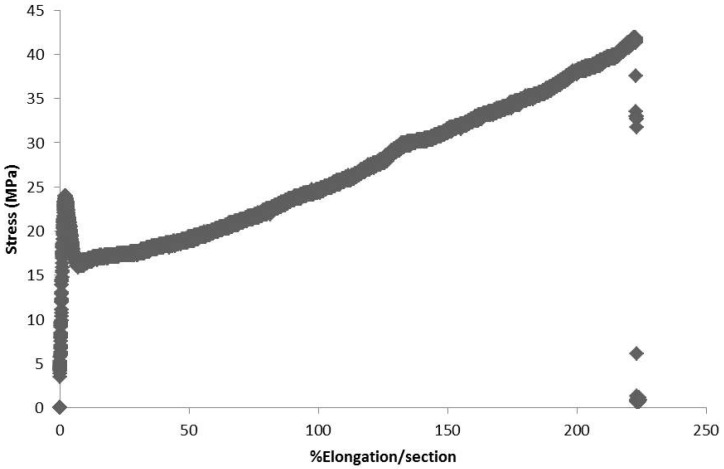
Example of a diagram obtained from the tensile test of the packaging.

Table 4 reports the mean data obtained, with an illustrative diagram of the stress/strain profile curve of the packaging at the beginning of the study is reported in Figure 1.

Empty containers (t0 samples) presented the highest value of tensile strain at yield with respect to treated samples.

**Figure 2:**
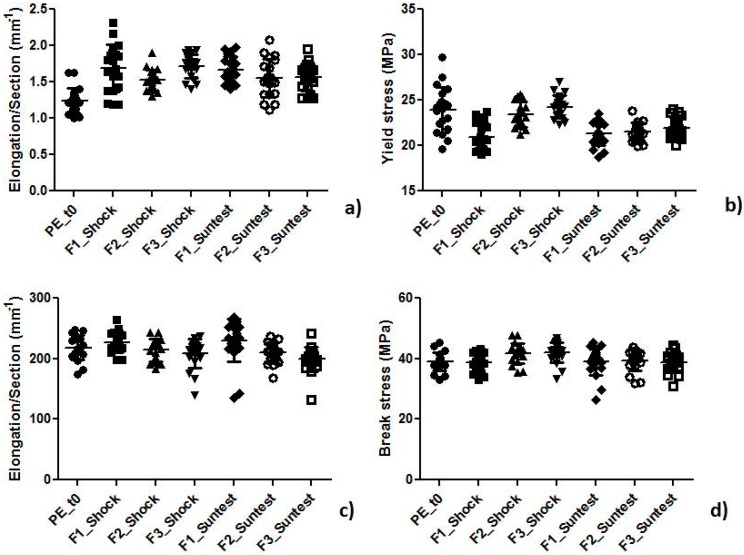
Trend of (**a**) yield strain; (**b**) yield stress; (**c**) break strain; and (**d**) break stress values obtained from all samples.

The trend for all values obtained from mechanical analysis (Figure 2) and the summary table (Table 5) reporting the significance values (*p*) of the statistical analysis performed by the Mann-Whitney test with a 95% confidence interval are reported. As can be observed from the table, significant changes occurred in the yield region of the curve after both treatments, especially after the sun irradiation simulating test. In this first part of the curve the polymer behavior is changing from an elastic to a plastic one. In particular, the sun irradiation simulating test modified, in a significant way, the polymer structure at this point, affecting the ability of the material to stretch and then return to its initial state. This result is very important because sunscreen products are subjected continuously to solar irradiation during their “in use” life. The effect of the treatments on polymer structure is not so evident in the break region, possibly due to this phenomenon occurring after a much longer time (almost 10 min of testing) and in this time the material fibers have time to regroup and act as the untreated material. These results explain that although the treatments do something, the change is not so great as to change their ability to resist breakage. 

#### 4.1.2. Colorimetric Analysis

Bottles before and after treatments were also analyzed from a colorimetric point of view, in order to discover possible changes in the color of the external part of containers. This type of analysis is very important for the quality of the final product, but also in order to establish its safety; in fact, the change in the packaging color, especially after solar irradiation, can indicate the formation of new chemical entities that could migrate into the formulation, affecting its safety for the final consumer. These considerations are important especially if the sunscreen product is intended for children or for those with sensitive skin.

For the colorimetric evaluation of packaging, the measurement of the yellowness index (YI) was performed. For this purpose, the colorimeter was used in order to obtain the three tri-stimulus values X, Y, and Z, of a color in accordance with the CIE system. Using the equation reported in ASTM 313, the yellowness index was calculated.

**Figure 3:**
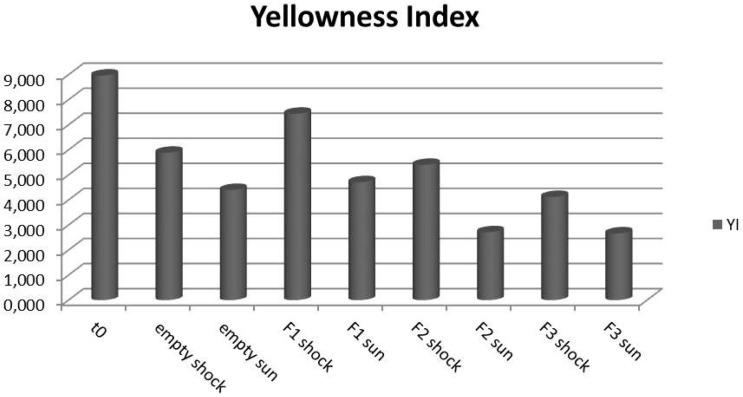
Yellowness index values obtained for the packaging.

Table 6 and Figure 3 report the yellowness index (YI) values with, and the related difference between, t0 and treated samples (ΔYI). 

Observing the yellowness index-calculated values, it is noted that all treated samples reported a decrease in yellowness index, with respect to those not treated and the empty sample (t0). The YI is a number that indicates the degree of departure of an object color from colorless or from a preferred white toward yellow. By this calculation, positive (+) ΔYI indicates increased yellowness and negative (−) ΔYI indicates decreased yellowness or increased blueness [23]. From this consideration it is possible to conclude that all treated samples presented a shift toward blue, so a decrease in yellowness. In particular, the most affected samples from treatments were the containers that underwent the solar simulated irradiation, as radiations provoke the greatest degradative effect. However, for both treatments, and especially for irradiation, the largest variations in color were observed for containers filled with formulations containing UV filters with respect to placebo and the empty bottles (t0), showing that the presence of filters in content could mainly influence changes in color. 

#### 4.1.3. Testing of Extractable Substances

The organic extractable profile of the packaging material investigated (LDPE/HDPE mixture containers) was established via the HS-SPME extraction processes, following a method set up in a previous work on polyethylene, because it provided a complete insight of all of the predominant organic extractables for the analyzed material [28]. 

After subtraction of the extraction blank results from the sample results, and removal of the interfering peaks associated with bleeding of the GC capillary column or SPME fiber coating, a list of compounds released by the analyzed polymer was extracted by GC/MS.

**Figure 4:**
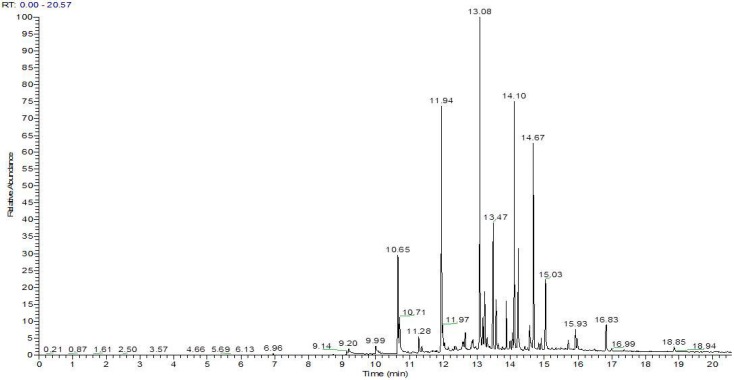
Chromatogram of the head space solid phase microextraction (HS-SPME) extract for containers.

The organic extractable profile of non-treated containers (t0) is summarized in Table 7 and the total ion current (TIC) chromatogram related to the GC/MS analysis of the obtained extract is shown in Figure 4.

These organic extractables generally fall into classes of compounds linked to the major constituents of the original plastic materials. For example, the profiles included compounds, such as antioxidants and additives (e.g., 2,4-Di-t-butyl phenol, phthalates), associated with the initial ingredients, impurities related to processing (e.g., esters), and degradation products of the polymers (aliphatic hydrocarbons). Once containers have been characterized at t0 (not treated), the next step was the characterization of the substances that could be extracted from containers filled with the formulations described above after treatment with solar irradiation and after undergoing thermal shock. 

**Figure 5:**
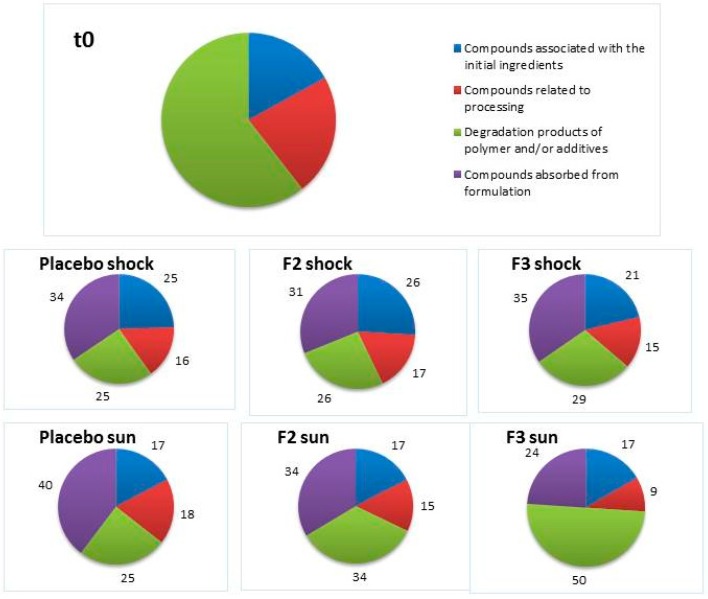
Percentage of areas of substances extracted from containers filled with formulations after treatments.

Figure 5 show graphs reporting the percentages of each class of substance extracted from containers filled with formulations (placebo, F2, and F3) and treated in comparison with samples at t0. All samples were extracted with the selected test method (HS-SPME) and the extracts were analyzed by GC/MS.

Data indicated that the largest percentage of compounds extracted from containers at t0 is associated with polymers and/or additives and degradation products. 

As can be seen for the material in contact with the formulations after UV-VIS irradiation and thermal shock cycles, substances closely related to the formulation were detected in relatively large amounts. These substances were identified as residues of C12-15 alkyl benzoate and 2-phenoxyethanol for all three formulations, and octocrylene only for F2 and F3 samples, representing an interval between 24% and 40% of the total extracted compounds.

Samples treated with simulated solar irradiation presented the largest variations in relative percentages of extracted compound classes. 

Samples of each formulation (300 mg) in contact with packages and after having undergone UV–Vis irradiation and thermal shock cycles have been analyzed by HS-SPME/GC-MS. No substances related to the polymeric materials were detected within the formulations.

### 4.2. Formulation Characterization

Twenty-four hours after preparation, 24 h after the end of treatments, and three months after treatments, all formulations were characterized from an organoleptic and rheological point of view.

Regarding the organoleptic characterization of formulations (color, odor, general aspect) no changes were observed after treatments and after three months of aging. Additionally, regarding pH measurements, no significant changes were revealed for the analyzed samples, as shown in Table 8.

The F3 formulation showed a lower pH than the placebo and F2 because of the presence of encapsulated filters in the suspension, which had a pH of 3.8–4.2.

Regarding the evaluation of viscosity, with respect to the index of rheological behavior of the formulations, Table 9 reports viscosity values expressed in mPas corresponding to a shear rate value of 10 rpm as representative of the entire curve obtained.

As the previous table shows, the placebo formulation presents lower viscosity values highlighting the presence of UV filters, both free and encapsulated, which increases the viscosity of the formulations. For the placebo, no significant changes were found after treatments, even after three months of storage.

Formulation with free UV filters (F2) showed no changes after treatments, but a decrease in the viscosity for all samples after a natural aging (three months) was observed. Instead, formulation with encapsulated filters (F3) presented an increase in viscosity after thermal shock cycle treatment, which remained the same after three months of storage. This result could be caused by the presence of the rheological agent PVP in the dispersion of encapsulated filters. 

### 4.3. Assessment of UV Filter Content

In order to monitor the content of UV filters in time and after treatments, spectrophotometric analyses were performed. It is well known from the literature that the simultaneous determination of two similar substances is not easy by direct UV spectrophotometry because of the spectral overlap of the main maxima [35]. Usually, a method based on the use of the first derivative of the ratios of the spectra is employed [36]. In this work the accuracy of the UV spectrophotometric method was demonstrated by the simultaneous determination of avobenzone and octocrylene by the high-performance liquid chromatographic method described in the literature and suitably modified [29]. The two substances were well separated by HPLC, showing retention times of 6.7 min and 9.7 min for Octocrylene and Avobenzone, respectively. Specific calibration curves for Octocrylene and Avobenzone as a mixture were constructed in a concentration range of 15–60 µg/mL and 5–20 µg/mL, respectively obtaining a very good separation and an high linear correlation coefficient (*R*^2^ > 0.99) By comparison with UV analysis the results are in good agreement (>95%). For these reasons spectrophotometric method was subsequently used 

The results are in good agreement. For these reasons the spectrophotometric method was subsequently used 

The calibration curves for avobenzone and octocrylene presented the following equations:

For Avobenzone: *y* = 0.0644*x* + 0.1071 (*R*^2^ = 0.9919)

For Octocrylene: *y* = 0.0262*x* + 0.0484 (*R*^2^ = 0.9963)

Using these equations, the amount of UV filters in the formulation was evaluated. The matrix influence was evaluated by analyzing the placebo absorbance at 303 and 361 nm (λ_max_ of octocrylene and avobenzone). The results demonstrated that no ingredient of formulation absorbs at the same wavelengths of the filters. 

Subsequently, the capability of the filter extraction method was investigated by using different dispersions of formulations in solvent (ethanol) in a wide range of concentrations of the two filters. The method demonstrated being able to extract almost the entire amount of filters present in formulations, included in a range of 95–105%. For monitoring the content of UV filters in formulations after the stress treatments stock solutions were prepared in triplicate.

Formulations were analyzed after treatments and after three months of storage (treated and untreated), withdrawing the formulations’ samples from two different parts of the containers, the upper part and the bottom, in order to reveal possible changes in filter amounts due to separation and/or stratification phenomena.

Table 10 reports the results of the spectrophotometric analysis, expressed as a percentage of the content of filters in an untreated formulation (t0 = 100%).

Considering an acceptability range for filter recovery of 90–110%, as observed from the table there are no significant changes for the amount of filters in formulation F3, neither after treatment nor after three months of storage.

Regarding formulation F2, there is a higher percentage of octocrylene recovery in the upper part of container after three months; this could be due to a water evaporation over time, or to migration of filters or separation phenomena, indicating the beginning of instability behavior of formulations containing free UV filters.

### 4.4. Near-Infrared (NIR) Analysis

The NIR (near-infrared) spectroscopy technique was used for a further non-destructive characterization of formulations in order to reveal possible instability phenomena before and after treatments. 

All samples were analyzed in triplicate by MicroNIR and data were evaluated using principle component analysis (PCA) on pretreated spectra, as previously explained. This technique was able to confirm the results obtained by the spectrophotometer analysis.

**Figure 6:**
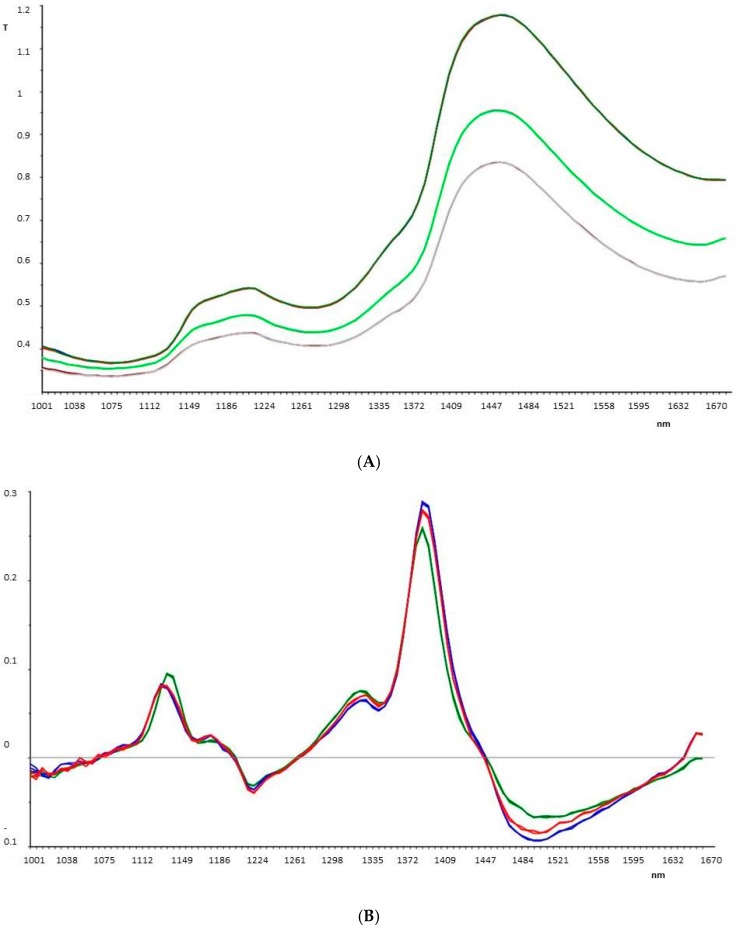

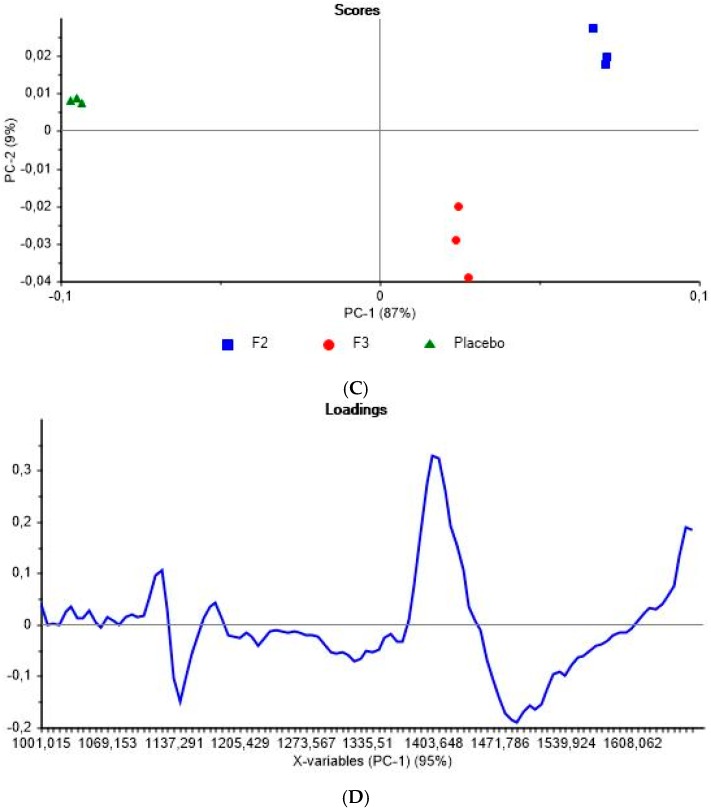
(**A**) Spectra of placebo sample (dark green) F3 (green) and F2 (purple line); (**B**) pretreated spectra of placebo sample (green line); F3 (red line); F2 (blue line); (**C**) Principal components analysis (PCA) plot; and (**D**) loadings plot, for placebo, F2, and F3 samples.

Firstly, as observed from Figure 6, this technique demonstrated the ability to distinguish the different formulations (placebo, F2, and F3).

In fact, the samples are distinct and separated from each other; in particular, the placebo is well separated along PC-1, while the two formulations that contain UV filters are closer together, but anyway differentiated along PC-2. 

**Figure 7:**
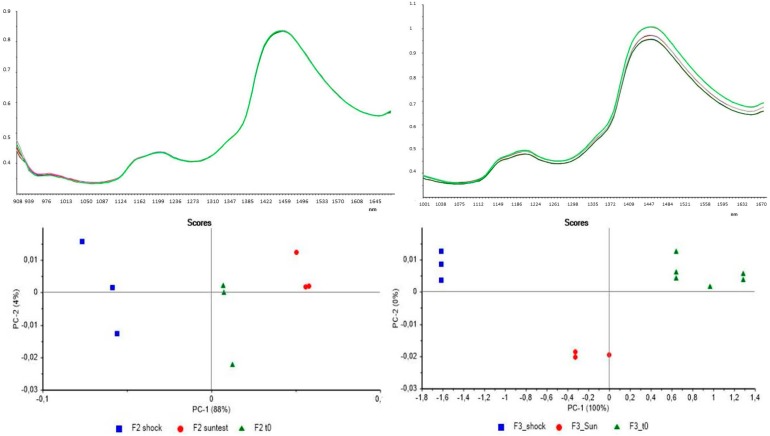
(**left**) up: Spectra of untreated F2 sample (dark green line) F2 after shock treatment (green line) and F2 after suntest treatment (red line); down: PCA for treated and untreated F2; (**right**) up: Spectra of untreated F3 sample (dark green line) F3 after shock treatment (green line) and F3 after suntest treatment (purple line); down PCA for treated and untreated F3.

The second step expressed the comparison between the untreated and the treated formulations in order to reveal possible changes due to treatments in the plastic packaging. Figure 7 shows the results of PCA performed on F2 and F3 formulations before and after UV irradiation (suntest) and thermal shock cycles (shock).

As observed from Figure 7, along PC-1, the samples are distinct from each other, to indicate a difference between the sample at t0 and the samples which underwent the two treatments. This consideration can be done both for the F2 and F3 formulations. It can be concluded than the two treatments caused some changes in both formulations, which reveals that the samples are different from each other. 

**Figure 8:**
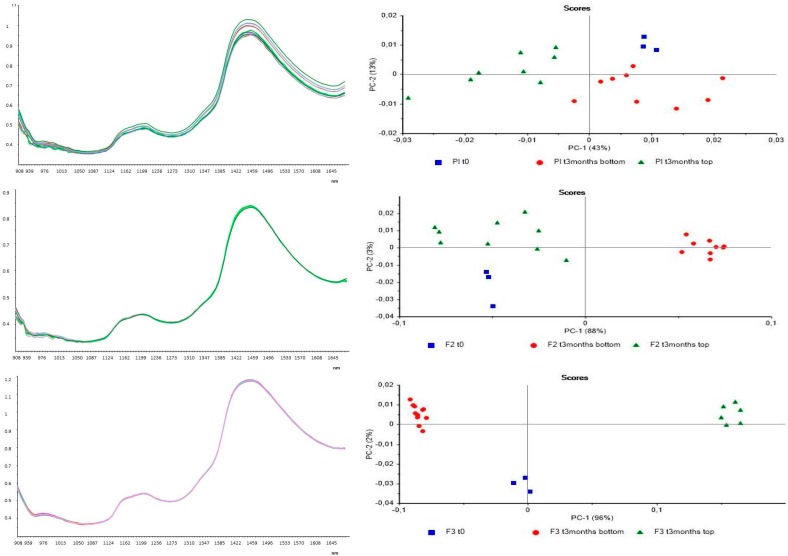
Left spectra and right PCA of from the top: placebo t0 (first three lines from above) and after three months, top (three lines in the center) and bottom (three lines below); F2 t0 (green line) and after three months, top ( blue line) and bottom (purple line); F3 t0 (purple line)and after three months, top (red line) and bottom (green line).

The last step was to compare each formulation at t0 (not treated) with the same after three months of natural aging; the samples after three months were withdrawn from two different positions of the containers, the top and the bottom, in order to consider possible changes in formulation structure due to stratification, creaming, or sedimentation phenomena, as shown in Figure 8.

As it can be observed from these graphs, the placebo does not present differences between the sample at t0 and after three months at both positions of container. Different considerations can be made instead for samples of F2 and F3 formulations. In fact, for F2 the NIR analysis revealed a difference between the sample deriving from the bottom and the one deriving from the top of container after three months of storage. The sample at t0 is, instead, comparable and not distinguished from the sample withdrawn from the top after three months. For formulation F3, this analysis revealed a difference between all samples, as the PCA sharply separated the three withdrawals. These results confirmed the analyses performed by the spectrophotometer on the filter content that some changes in the formulations occur over time.

### 4.5. Multiple Light Scattering Analysis

**Figure 9:**
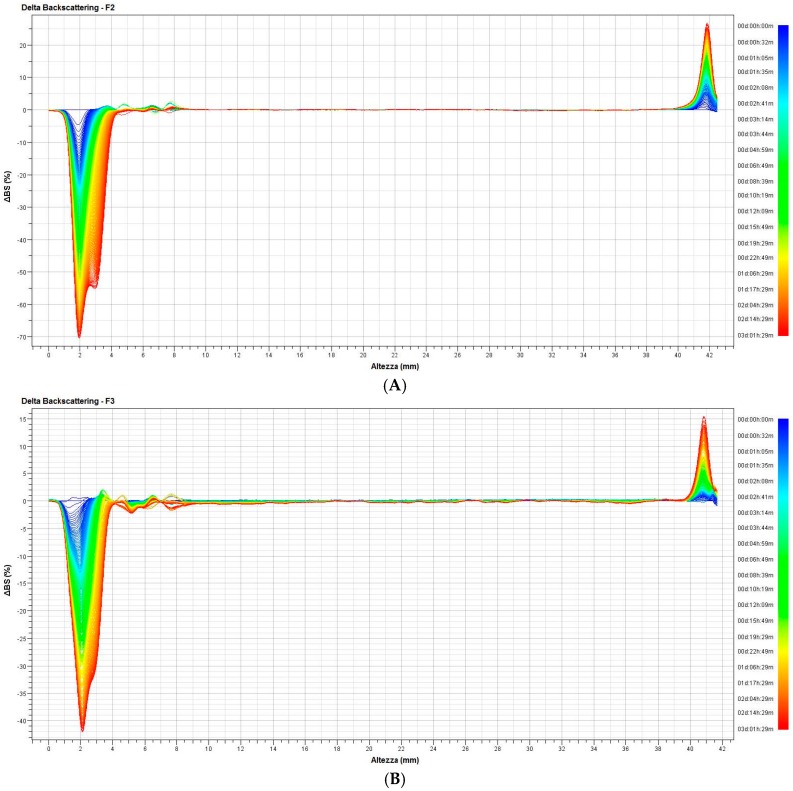
ΔBS (%) of (**A**) F2 and (**B**) F3 samples.

The multiple light scattering technique was used in order to evaluate the stability of formulations and to confirm the results obtained with other techniques. In particular, NIR analysis and spectrophotometric evaluations on F2 and F3 revealed a difference between samples withdrawn from the top and the bottom of containers, as there were some instability phenomena (creaming, stratification, etc.) after three months of stabilization. The ΔBS graphs (%), the TSI (Turbiscan stability index) values, and the kinetics of separation are reported in Figure 9. ΔT values are not shown because the samples were milky and, therefore, there are no variations in transmission. 

As seen in Figure 9, sample F2 showed a clear and significant clarification for the bottom sample (70% ΔBS). Simultaneously, from the upper portion of the vial, there was, instead, a moderate creaming phenomenon (+25% ΔBS). Observed clarification and creaming phenomena are indicative of an instability of the sample, since values are outside the range of ±10% ΔBS.

Contrary to sample F2, for sample F3, although it exhibited an instability profile similar to the previous one, variations of ΔBS (%) from the bottom and top portions are markedly different: −40% vs. −70% (clarification) and +15% vs. 25% (creaming), respectively.

The differences must be considered as referring to samples held at room temperature for three months.

**Figure 10:**
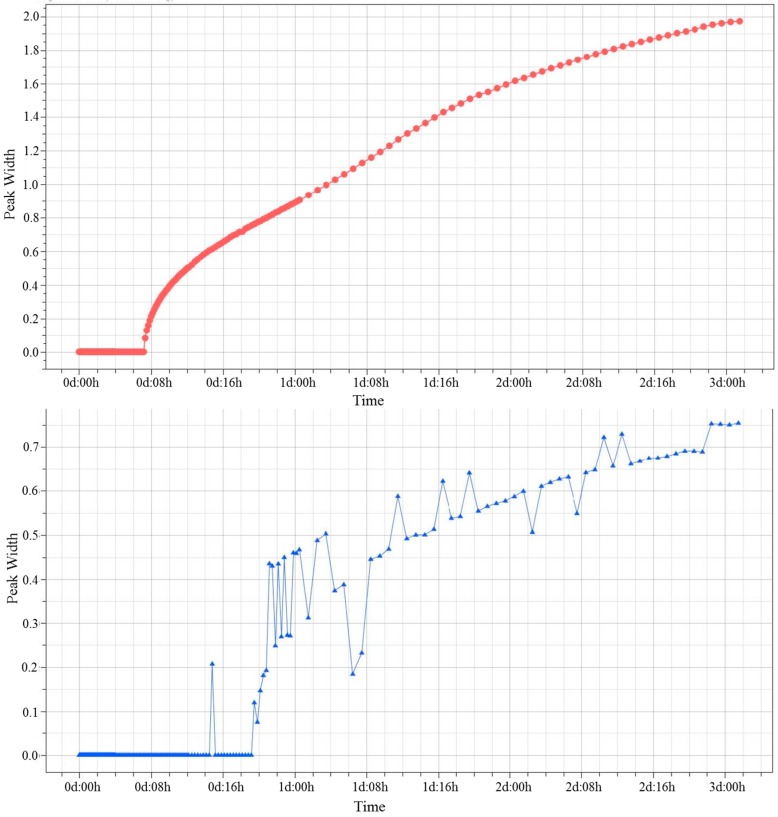
Peak’s width (BS) of F2 (**top**) and F3 (**bottom**) samples.

In Figure 10, the kinetic profile of the creaming phenomenon for both formulations is shown.

For each curve, the slope was calculated by software for three time intervals: from 6 to 12 h, from 12 to 48 h, and from 48 to 72 h. The first segment of curve F2 showed a high kinetic rate of creaming, with a slope value of 1.67 mm/d; the formulation F3 showed no instability phenomena (slope = 0) until about 20 h. Thus, this formulation was demonstrated to be more stable than F2 for the first part of the study. The second portion of the curve presented a slope for F2 of 0.16 mm/d, while F3 started to show a creaming phenomenon, with a slope = 0.32 mm/d.

**Figure 11:**
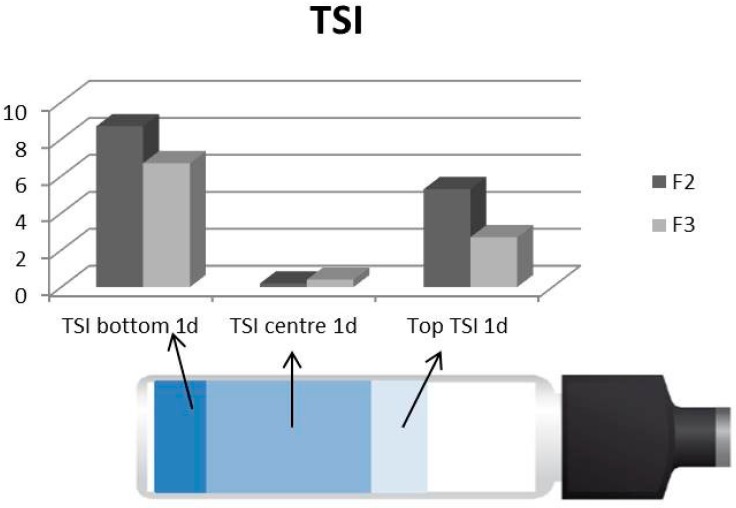
TSI for F2 and F3 at day one for the bottom, center, and top of the vial.

For both curves, the third portion seemed to nearly reach the plateau, with a slope for F2 equal to 0.06 and for F3 equal to 0.19 mm/d. Finally, the TSI values are reported for both formulations. The TSI at one day for the three parts of the container (bottom, center, top) is reported in Figure 11, while Table 11 reports the values of the global TSI for both formulations at day one, two, and three.

The TSI for the different positions of the vial indicates that the bottom is the zone that presents the highest instability (clarification phenomenon) because of the highest value of TSI. The central part does not present instability phenomena (lowest values of TSI), confirming the absence of aggregation phenomena. Formulation F2 was revealed to be more unstable than F3 both for the top and bottom zones; in fact, clarification and creaming phenomena are more pronounced. 

The global TSI profiles, for the top and bottom, only reconfirm what has already emerged from the ΔBS profiles (%): formulation F3 is more stable than formulation F2, proving to separate more slowly during the three days of analysis. This confirms the different stratification previously found by other techniques.

## 5. Conclusions

This work aimed to study the stability of a sunscreen product, packed in a bottle made of a very commonly used LDPE/HDPE mixture, in conditions simulating the possible stress conditions that solar products could meet during their “in use” life in order to assure quality, but also safety, of the final product. In fact, the results reported in this work demonstrated clearly that some stress conditions that sunscreen products are exposed to during their “in use” life can affect both quality and safety of the product. Interesting results were obtained from the analyses of plastic packaging (made by a mixture LDPE/ HDPE) used as a primary pack for the formulations. Firstly, all of the pack underwent significant changes in its elastic/plastic behavior, especially after solar irradiation. This result is very important because these products are subjected continuously to solar irradiation during their “in use” life.

Furthermore, treated containers also presented some changes in external color (yellowness index variations), more accentuated for samples treated with simulated solar radioactivity. These results underlined that some changes in chemical composition can occur in the pack and these modifications can lead to the formation of unknown substances in the formulation. Finally, from the evaluation of the extractable profile of untreated and treated packaging materials, a possible absorption of some ingredients of the formulations were shown. In particular, substances closely related to the filling formulation were detected; these substances were identified as residual of C12-15 alkyl benzoate, 2-phenoxyethanol for all three formulations, and octocrylene only for F2 and F3 samples. These results highlighted clearly that a reduction of the UV filter in the formulation can occur over time, reducing the protective effect of the product when applied to the skin. 

All of these considerations on packaging are very important, especially if the sunscreen product is intended for children or for those with sensitive skin. Regarding the characterization of formulations, no changes in pH or organoleptic properties were revealed and no significant differences were obtained by rheological analysis. 

Regarding the chosen UV filters butyl methoxydibenzoylmethane (avobenzone) and octocrylene, in free and encapsulated forms (sol-gel silica glass; commercially available product), the simultaneous use of NIR and multiple light scattering revealed the instability of formulation F2 containing free-form UV filters. A difference between samples withdrawn from the top or from the bottom of containers was also revealed by NIR spectroscopy, confirming possible instability phenomena.

In fact, observing data obtained by Turbiscan analysis, it can be found that clarification and creaming phenomena are more pronounced in F2 than in F3, so the formulation containing encapsulated filters is more stable than the formulation with free UV filters. The evaluation of the UV filter amount indicates that there are no significant changes of the filter amount in formulation F3, neither after treatment nor over time. Regarding formulation F2, there is a trend towards a more concentrated filter in the upper part of the container after three months of storage. These results confirmed the multiple light scattering analyses with a tendency of the filter to separate. 

Results obtained from this work permit the conclusion that the LDPE/HDPE mixture chosen for packaging is not suitable for sunscreen products; the solar simulator irradiation is the essential test to evaluate the stability of this kind of product. Furthermore the use of NIR and multiple light scattering techniques can be promising to assess short-term changes hardly detectable with other techniques commonly used in cosmetics.

In conclusion, this study confirms the importance of studying all aspects related to a final product. Although the evaluation of formulation is important, it is also essential to verify the use of suitable packaging in order to assure the quality, the efficacy, and also the safety of the final product.

## Figures and Tables

**Table 1:** Percentage composition of the formulations.

Phase	Ingredient	INCI	F1	F2	F3
A	Water	Aqua	83.9	71.9	53.9
	Sucrathix VX	Microcrystalline cellulose, cellulose Gum, and Xanthan Gum	1	1	1
	Glycerin	Glycerin	2	2	2
	Eusolex UV Pearls OB SX	Aqua, octocrylene, sorbitol, butyl methoxydibenzoylmethane, silica, PVP	-	-	30
	Disodium EDTA	Disodium EDTA	0.1	0.1	0.1
B	Montanov L	C14-22 Alcohols (and) C12-20 alkyl glucoside	1	1	1
	Montanov 82	Cetearyl Alcohol (and) coco-glucoside	4	4	4
	Eusolex 9020	Butyl methoxydibenzoylmethane	-	3	-
	Eusolex OCR	Octocrylene	-	9	-
	Tegosoft TN	C12-15 alkyl benzoate	7	7	7
C	Verstatil PC	Phenoxytehanol, caprylyl glycol	1	1	1

**Table 2:** Operating parameters for gas chromatography-mass spectrometry (GC/MS) analysis.

Column	Restek Capillary Column Rtx-5MS 30 m × 0.25 mm ID × 0.25 µm
Oven Program	Start 60 °C, hold for 4.5 min; ramp 20 °C/min to 280 °C, hold for 5 min
Injector	PTV Splitless 250 °C; Splitless time 4.5 min
Carrier Gas	He, 1 mL/min constant flow
MS Transfer line temperature	270 °C
MS Detection details	70 eV (+EI); Ion source 250 °C; Mass range 50–650 amu; scan rate: 870 amu/s

**Table 3:** Micro near-infrared (MicroNIR) instruments’ parameters.

Illumination Source	Two Integrated Vacuum Tungsten Lamps
Illumination geometry	Flood illumination/0° observer
Input aperture dimensions	2.5 × 3.0 mm
Sample working plane	0–15 mm from window, 3 mm optimal distance
Detector	128 pixel InGaAs photodiode array
Pixel Size/Pitch	30 µm × 250 µm/50 µm
Wavelength range	950–1650 nm
Pixel to pixel interval	6.2 nm
Spectral bandwidth (FWHM)	<1.25% of center wavelength (1% typical)
Spectral in-band	LVF blocking >4 OD Average

**Table 4:** Tensile test mean data obtained for packaging.

Sample	Tensile Strenght (σM) = Yield Stress (σy) (MPa)	Elongation at Yield/Section (mm^−1^)	Tensile Stress at Break (σB) (MPa)	Elongation at Break/Section (mm^−1^)
T0 empty	23.954	2.099 × 10^−1^	39.228	37.079
F1 shock	20.960	2.870 × 10^−1^	38.418	38.568
F1 sun	21.268	2.827 × 10^−1^	39.074	39.184
F2 shock	23.415	2.587 × 10^−1^	41.783	36.387
F2 sun	21.524	2.635 × 10^−1^	39.490	35.625
F3 shock	24.249	2.918 × 10^−1^	41.958	35.396
F3 sun	21.941	2.661 × 10^−1^	38.879	33.853

S.D. < 10%; Legend: shock: samples subjected to thermal cycles test; sun: samples subjected to the photostability test.

**Table 5:** Statistical significance *p* values obtained from packaging analysis, summarized with one, two or three asterisks (*, **, ***) depending from the level of statistical significance.

Suntest					Shock			
Parameter	t0 vs. F1	t0 vs. F2	t0 vs. F3	F2 vs. F3	t0 vs. F1	t0 vs. F2	t0 vs. F3	F2 vs. F3
Yield stress	0.0002 ***	0.0007 ***	0.0040 **	0.3104	0.0001 ***	0.4735	0.5609	0.0658
Yield strain	<0.0001 ***	0.0003 ***	<0.0001 ***	0.7972	<0.0001 ***	<0.0001 ***	<0.0001 ***	0.0005 ***
Break stress	0.4094	0.2287	0.7972	0.2792	0.8604	0.0071 **	0.0023 **	0.7353
Break strain	0.0193 *	0.1264	0.0031 **	0.0601	0.1896	0.5075	0.2853	0.4735

**Table 6:** YI and ΔYI values of non-treated and treated packaging samples.

Sample	YI	ΔYI
t0	8.966	
Empty shock	5.886	−3.080
Empty sun	4.400	−4.566
F1 shock	7.442	−1.524
F1 sun	4.709	−4.257
F2 shock	5.402	−3.564
F2 sun	2.712	−6.255
F3 shock	4.120	−4.846
F3 sun	2.666	−6.301

**Table 7:** Organic extractables profile of non-treated containers made of low-density polyethylene (LDPE)/high-density polyethylene (HDPE) mixture.

Identification	CAS NR	Chemical Formula	Molecular Weight	HDPE % Area
p-Benzoquinone, 2,6-di-tert-butyl	719-22-2	C14H20O2	220	1.59
Diisopropylnaphtalene	-	C16H20	212	1.71
2-Propanol, 1-chloro, phosphate	13674-84-5	C9H18Cl3O4P	326	5.76
Diisobutyl phthalate	84-69-5	C16H22O4	278	2.61
7,9-Di-tert-butyl-1-oxaspiro(4,5)deca-6,9-diene-2,8-dione	82304-66-3	C17H24O3	276	2.24
Octinoxate	5466-77-3	C18H26O3	290	1.00
Diisooctyl phthalate	131-20-4	C24H38O4	390	1.57
Squalene	111-02-4	C30H50	410	0.71
Siloxanes	/	/	/	18.65
Aliphatic hydrocarbons	/	/	/	34.29
Olefins	/	/	/	29.87

**Table 8:** pH values of formulations before and after treatments and after three months.

Sample	t0	Suntest	Shock	t3 Months	Suntest Three Months	Shock Three Months
Placebo	5.65	5.66	5.51	5.58	5.55	5.57
F2	5.49	5.70	5.47	5.52	5.48	5.50
F3	4.61	4.65	4.64	4.62	4.62	4.63

**Table 9:** Viscosity values of formulations before and after treatments and after three months.

Shear Rate: 10 rpm					
Placebo	η (mPas)	F2	η (mPas)	F3	η (mPas)
t0	5600	t0	9200	t0	9000
Suntest	5800	Suntest	8800	Suntest	9200
Shock	6800	Shock	9000	Shock	11,800
t3months	5600	t3months	7800	t3months	9600
Suntest threemonths	5000	Suntest threemonths	7600	Suntest threemonths	9000
Shock threemonths	5600	Shock threemonths	7800	Shock threemonths	12,400

**Table 10:** Filters in formulations with relation to untreated formulations (t0).

**F2**									
**Filter**	**t0**	**Sun**	**Shock**	**t3months Top**	**Suntest Three Months Top**	**Shock Three Months top**	**t3months Bottom**	**Suntest Three Months Bottom**	**Shock Three Months Bottom**
OCR	100.00	99.64	101.25	113.32	110.59	102.88	102.26	103.14	103.77
AVO	100.00	98.92	99.39	109.57	107.29	101.31	98.50	101.94	102.04
**F3**									
**Filter**	**t0**	**Sun**	**Shock**	**t3m Up**	**t3m Sun Up**	**t3m Shock Up**	**t3m Bottom**	**t3m Sun Bottom**	**t3m Shock Bottom**
OCR	100.00	94.81	92.37	102.27	107.06	101.88	100.59	99.85	99.37
AVO	100.00	94.68	95.42	100.05	103.96	99.97	97.91	96.57	97.39

**Table 11:** Global TSI for F2 and F3 at days one, two, and three.

Measure	Global TSI 1 day	Global TSI 2 day	Global TSI 3 day
F2	3.3	4.3	5.1
F3	2.3	3.1	3.5
